# Investigation of synthesis time and type of seed along with reduction of template consumption in the preparation of SAPO-34 catalyst and its performance in the MTO reaction

**DOI:** 10.1039/d0ra05673a

**Published:** 2020-09-17

**Authors:** Sahar Akhgar, Jafar Towfighi, Marzieh Hamidzadeh

**Affiliations:** Chemical Engineering Department, Tarbiat Modares University P.O. Box 14115-143 Tehran Iran Towfighi@modares.ac.ir +982182883311; National Petrochemical Company, Petrochemical Research and Technology Company P.O. Box 1435884711 Tehran Iran

## Abstract

SAPO-34 catalysts were synthesized through the seeding approach under different seed conditions. The different seed synthesis times (6 h, 12 h, and 24 h) and three types of seeds were evaluated: the dried seed, the calcined seed, and the mother liquor from an unseeded synthesis, called the solution seed. Pure SAPO-34 was obtained using 12 h and 24 h solution seeds, in which a 40% reduction of template consumption was achieved simultaneously. All seeding induced samples represented higher catalytic performance in the MTO process than conventional SAPO-34 due to the smaller crystallite/particle sizes and larger external surface areas and mesopore volume. Furthermore, the changes in the acidity of samples affect their performance. The maximum olefin selectivity under industrial feed conditions (72 wt% methanol in water) was 91.79% for the sample prepared from the 12 h solution seed, which was 14.43% higher than the unseeded sample. Although this sample did not have the longest lifetime, it showed a 330 min lifespan, which was at least twice more than that of the conventional one (150 min). The sample prepared from the 6 h solution seed showed the longest lifetime of more than 500 min among all catalysts, although it was contaminated with a little SAPO-5.

## Abstract 2

SAPO-34 catalysts were synthesized through the seeding approach under different seed conditions.

## Graphical abstract

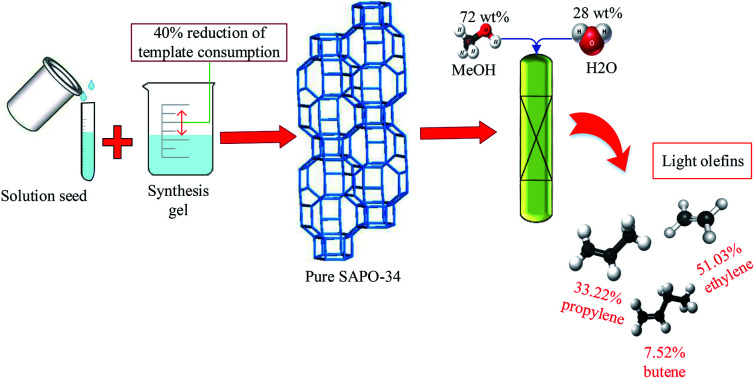

## 1. Introduction

Ethylene and propylene are the primary building blocks in the chemical industry and are mainly produced by fluid catalytic cracking (FCC) and steam cracking. The rising demand for these materials has recently created a gap between supply and demand, especially for propylene. In addition, these processes face serious challenges such as low yield of light olefins, high crude oil prices, environmental pollution, and high energy consumption. Therefore, in recent decades, methanol conversion to light olefins (MTO) has been considered as an alternative method that can produce light olefins from the non-oil route.^1–4^

Catalysts are necessary components of many industrial processes (*e.g.*, two-dimensional materials that have become an important platform to design and synthesize single-site catalysts and have wide applications in a series of reactions such as CO_2_ reduction, CO oxidation, *etc.*).^5–8^ Up to now, among all catalysts, SAPO-34 zeolite with a chabazite (CHA) framework has shown the best proficiency in the MTO process because of its high shape selectivity, small pore size (0.38 nm), mild acidity, large accessible surface area, and hydrothermal stability.^9–11^

The preparation of pure SAPO-34 is important both in terms of the characterization and the application points of view. According to the synthesis conditions, SAPO-34 with the CHA structure competes with SAPO-5 phase (AFI structure), as the CHA structure increases with increasing the template amount.^12,13^ The most ordinary template applied for the synthesis of SAPO-34 is tetraethylammonium hydroxide (TEAOH), but high price restricts its usage.^14^ Barot *et al.*^15^ reported that pure SAPO-34 with small crystal sizes was procured at the TEAOH/Al_2_O_3_ molar ratio of more than 2, while with less than this amount, SAPO-5 and SAPO-18 were obtained as an impurity. A combination of different amines has been developed to dominate the high cost of some toxic templates.^16^

In general, templates as structure-directing agents (SDAs) have some disadvantages as follows: (1) templates are expensive; thus the end product is expensive, (2) high-temperature combustion is required to remove organic templates, which leads to the formation of dangerous gases, including carbon dioxide and NO_*x*_, (3) using an SDA creates a considerable amount of contaminated water that is not eco-friendly, (4) the released energy from burning templates can be very harmful to the inorganic framework of catalysts. The mentioned disadvantages surely reduce the commodious usage of the templates.^17^

The main role of SDAs in the synthesis of crystals is to form zeolite nuclei. To synthesize zeolite catalysts, researchers have recently applied two strategies to create nuclei in primary aluminosilicate gel. The first method is to use zeolite crystals as seeds, and the second method is to use zeolite solution as seed. Zeolite crystals can be either in the dried form or in the calcined form ref. 17. Sun *et al.*^14^ synthesized nano-sized hierarchical SAPO-34 samples *via* a seed assisted method using different concentrations of triethylamine (TEA). Due to the use of seeds, SAPO-34 without any impurity was gained with a TEA/Al_2_O_3_ molar ratio of 2. Furthermore, they were able to reduce the amount of organic template by 40%.

Generally, using seeds resulted in (1) increasing crystallization rate, (2) decreasing the formation of an undesirable phase, (3) decreasing the synthesis time, and (4) controlled the particle size.^18,19^ Sun *et al.*^20^ succeeded in synthesizing pure SAPO-34 through a seeding-induced method applying TEA as the sole template. In comparison to the micron-size SAPO-34, the hierarchical nanoparticle samples had 4-times higher lifespan and showed selectivity of 85% to ethylene and propylene. Chen *et al.*^21^ constructed SAPO-34 nanoparticle zeolites with morpholine as the only template by applying a nano-seed inducing method. The best-prepared sample demonstrated 4-times higher lifespan (166 min) and selectivity about 5% higher than the conventional micron-sized SAPO-34 (46 min). In a recent study, Sun *et al.*^22^ synthesized SAPO-34 samples applying MOR-treated micrometer SAPO-34 as seed crystals. The resultant samples with a reduction of crystal size to 200–500 nm and lower acidity, as well as larger external surface areas, showed better performance in the MTO reaction compared to the conventional sample. In another research, Lu and coworkers^23^ synthesized pure SAPO-34 using activated seed crystals with a low concentration of template (MOR/Al_2_O_3_ = 1). The results showed that the addition of seed crystals not only decrease the crystal size to 500–800 nm but also led to an appropriate acidity. The best sample indicated a lifetime of about 140 min. Lyu *et al.*^24^ added different amounts of calcined SAPO-34 as a seed to synthesize SAPO-34 *via* dry gel conversion method using a low dosage of template (TEAOH/Al_2_O_3_ = 1). The obtained SAPO-34 with a crystal size of around 25 nm showed 83.6% selectivity toward light olefins. According to the results of previous researches, it is still necessary to synthesize a sample with considerable reduction of template consumption along with an excellent catalytic performance.

In conclusion, using an organic template increases the cost of synthesis and the pollution of the environment. Therefore, it is still necessary to reduce the consumption of templates to enhance the SAPO-34 application. In this study, the seeding method was used in the synthesis of SAPO-34 using three types of seeds (solution, dried and calcined seed) and three different seed synthesis times (6 h,12 h, and 24 h), to decrease the template consumption without significant impact on the zeolites crystallinity and their catalytic performance. As a result of the changes, the template consumption was reduced by 40% in this study. To the best of our knowledge, it is the lowest amount of mixed templates has been reported and deployed to synthesize the pure SAPO-34 until now. Also, there has been no report about the effects of seed synthesis times and different types of seeds on the preparation of SAPO-34 up to now.

## 2. Experimental

### 2.1 Materials

Aluminum isopropoxide (AIP), silica gel (SiO_2_), and phosphoric acid (H_3_PO_4_, 85% wt), were used as the source of Al, Si, and P, respectively. Tetraethylammonium hydroxide (TEAOH, 20% wt in water) and morpholine (MOR) were used as microporous templates.

### 2.2 Preparation of SAPO-34 zeolite seeds

SAPO-34 seeds were prepared with a molar composition of Al_2_O_3_:P_2_O_5_:0.6SiO_2_:2TEAOH:70H_2_O at 200 °C. In the first step, phosphoric acid and water were mixed. Then, aluminum isopropoxide and template were added gradually during 2 h. After 1 h, Si source was added to the preparation system and then the resultant gel stirred continuously overnight at room temperature. Before transferring the materials into the autoclave, the ultrasonic operation (output power of 300 W for 30 min) was performed to reduce the crystal sizes and to increase the uniformity. Then the gel was poured into a 60 ml autoclave and was heated at 200 °C for 6 h, 12 h, and 24 h. The mother liquor was put out of the autoclave and was used as the solution seed. Following the centrifugation and washing with deionized, the solid product was dried at 110 °C for 10 h to obtain a dried seed. The resultant solid product was calcined at 550 °C for 5 h to remove the organic templates and was used as a calcined seed.

### 2.3 Preparation of seeding SAPO-34 samples

Seven samples with different seed conditions were prepared *via* a seeding-induced method using the molar composition of Al_2_O_3_:P_2_O_5_:0.6SiO_2_:(*x*)MOR:(*x*)TEAOH:70H_2_O:8 wt% seed at 200 °C for 24 h (*x* = 1, 0.8, 0.6). The resulting samples were named according to Table 1. The procedure of gel preparation was similar to the abovementioned process, except it did not undergo the ultrasonic operation, and the seed was added as the last material. For comparison purposes, a conventional sample (S1) was prepared with a molar composition of 1Al_2_O_3_:1P_2_O_5_:0.6SiO_2_:1MOR:1TEAOH:70H_2_O, without adding any SAPO-34 seed.

**Table 1:** The relative crystallinity, crystallite size, and phase purity of samples

Samples	Template amount (mol)	Seed conditions		Crystallite size (nm)	Relative crystallinity (%)	Product
		Hydrothermal time (h)	Type			
S1	1	—	—	92.7	100	SAPO-34
S2	1	6	Solution	60.4	88.06	SAPO-34
S3	0.8	6	Solution	76.6	87.49	SAPO-34
S4	0.6	6	Solution	91.8	—	SAPO-34 + SAPO-5
S5	0.6	12	Solution	55.8	67.98	SAPO-34
S6	0.6	24	Solution	66.7	76.08	SAPO-34
S7	0.6	12	Dried	46.1	—	SAPO-34 + ALPO_4_-18
S8	0.6	12	Calcined	38.5	—	SAPO-34 + ALPO_4_-18

## 3. Characterization

For the phase identification of samples, X-ray diffractions (XRD) were recorded on a Philips X'Pert MPD apparatus using Cu-Kα radiation (wavelength = 1.5417 Å) with a step size of 0.02° s^−1^. Field emission scanning electron microscopic (FESEM) images were carried out with a TESCAN MIRA3 device to determine the morphology of the catalysts. The average particle sizes were determined with ImageJ software. The device was also connected with an energy-dispersive X-ray spectroscopy (EDS) analyzer for measuring the surface chemical composition of the samples. Inductively coupled plasma mass spectrometry (ICP-MS) was performed by ELAN DRC-2 equipment (PERKIN ELMER SCIEX Company). N_2_ physisorption at 77 K using was performed on a QuantaChrome ChemBET3000 analyzer to obtain the textural properties of samples. The NH_3_-TPD measurements were conducted on 0.04 g of each catalyst placed in a reactor using a NanoSORDNS91 (made by Sensiran Co) device. Prior to starting each TPD run, the catalyst was degassed at 500 °C for 1 h, and then was cooled down to 110 °C. Following that, 5% NH_3_/He was adsorbed. Afterward, the sample was purged in a stream of helium at 110 °C for 30 min to eliminate loosely bounded ammonia. Eventually, the sample was heated up to 500 °C at a heating rate of 10°C min^−1^ in a He stream.

## 4. Catalyst testing

**Fig. 1:**
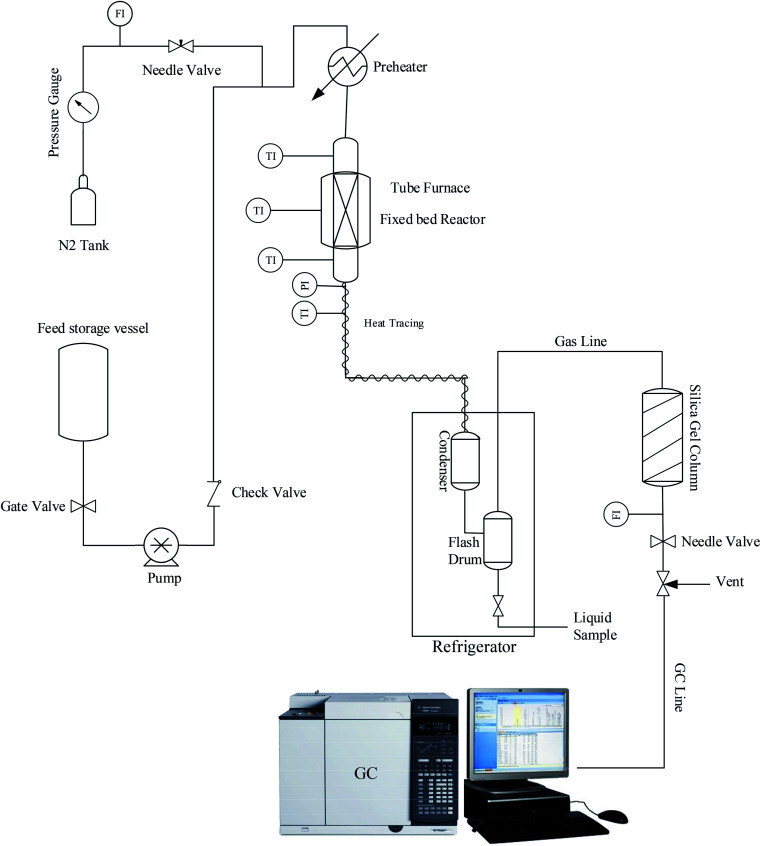
Experimental system for evaluation of prepared SAPO-34 catalysts.

Fig. 1 illustrates the lab setup used in this work. A certain amount of calcined samples (2 g, 20–40 mesh) with 4 g of SiC as the inert material were placed in the center of a catalyst bed to perform experimental tests under atmospheric pressure. For the initial preparation process, the nitrogen gas stream was passed through the catalyst bed at 550 °C for 1 h to remove any possible moisture or contamination. Then, the reactor temperature was set to 425 °C as the reaction temperature. When the bed temperature reached 425 °C, the feed stream containing a mixture of 72 wt% methanol in water was injected into the reactor, which gave the WHSV of 2 h^−1^. Reactor outlet products contain a wide range of hydrocarbons, water, and water-soluble oxygenated compounds that require an initial separation to determine them. Hence, heavier hydrocarbon compounds with water, methanol, and other soluble compounds were separated into the liquid phase after passing the condenser. Lighter products, including methane, ethane, ethylene, propylene, *etc.*, enter an online gas chromatograph (Agilent GC 7890A) to determine the percentage composition of the exhaust gas.

## 5. Results and discussion

**Fig. 2:**
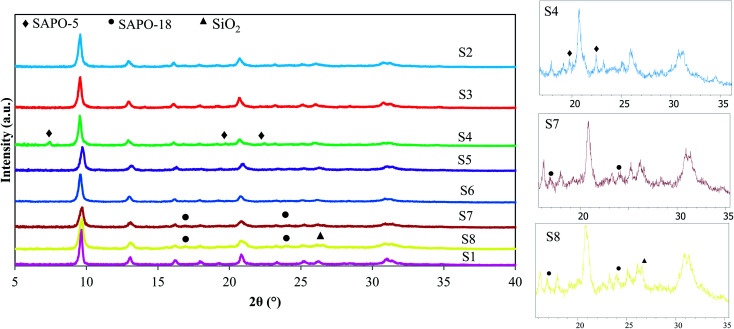
XRD patterns of calcined samples.

Fig. 2 illustrates the XRD diffractions of calcined catalysts. All prepared samples, except S4, S7, and S8, show the construction of pure SAPO-34 with a CHA topology matching with JCPDS card no. 00-047-0429. The relative crystallinity, which is determined according to the sum of peak intensities at 2*θ* = 9.5°, 13°, and 20.7°, and crystallite sizes are listed in Table 1 along with each sample phases. S1 with the highest crystallinity is used as the reference sample in relative crystallinity calculation. The differences of crystallite size as well as relative crystallinity between the samples arise from the different nature of seed synthesis conditions and the difference between the concentrations of used templates. The results show that by addition of seed, relative crystallinity decreases. The recrystallization of seed crystals during the hydrothermal synthesis may lead to lower crystallinity. In addition, the higher concentration of organic template resulted in the higher crystallinity, which is in accordance with Dargahi and coworkers^12^ findings. Therefore, the highest crystallinity of S1 is attributed to more amount of template and the absence of seed. The peak at 2*θ* = 9.5° is narrower for S1 than the rest of the samples, which confirms the higher crystallinity of this sample.

The S4 sample, prepared with 6 h solution seed, shows three peaks of SAPO-5 as an impurity (2*θ* = 7.46°, 19.73°, and 22.43°). It might be due to the inadequate seed synthesis time and the simultaneous reduction of template concentration. It suggests that the possible amorphous materials that existed in the seed synthesized for 6 h are thermodynamically unstable, in a way that some of them transform to the SAPO-5 phase during the crystallization of the S4 sample. With further increase of seed synthesis time, the SAPO-5 impurity disappears and the pure SAPO-34 phase is obtained. Using dried and calcined seeds shows peaks at 2*θ* = 16.9° and 23.9°. These peaks are related to the ALPO_4_-18 with an AEI structure, which is coexisted with the SAPO-34 phase.^25^ A very small peak can also be seen for S8 at a degree of about 26.5, which is the characteristic peak of SiO_2_. The peak broadening of this sample in the area between 2*θ* of 20°–35° can be related to amorphous materials. It might be deduced that the higher amount of template exists in the solution seed compared to the dried and calcined forms is more beneficial to increase the purity of the final phase.

The crystallite sizes of the samples that are calculated according to Scherrer's equation (eqn (1)) are reported in Table 1. *D* is crystallite size (nm), *λ* is X-ray wavelength equal to 1.5417 Å, *β* is the line broadening at half of the highest intensity (FWHM), and *θ* is Bragg angle. Sample S1 has the largest crystallite size among all prepared catalysts. It has been explained that the nucleation process in a seed-assisted synthesis can occur on the external surface area of seed crystals, which can act as active sites for nucleation in addition to the self-nucleation process that occurs on the solid–liquid interface of gel. In a non-seed system, such as S1, more times is required to form nucleus, which resulted in larger crystallite sizes.^26,27^ The results reveal that with increasing the amount of template, the crystallite sizes decrease. It can be justified that more template amounts cause an increase of nucleation rate in the solution, resulting in a large number of small crystals rather than a small number of larger crystals.^28^ Furthermore, the larger crystallite size of S4 among seeded-samples could be related to the incomplete structure of the added seed, which is prepared in a lower synthesis time. It seems that the crystal growth for S4 is started as the seed added to the reaction gel and led to larger crystallite size, while for S5 and S6, the recrystallization of the seed crystals with completed framework may occur and led to smaller crystallites as a result of slower crystal growth rate.1
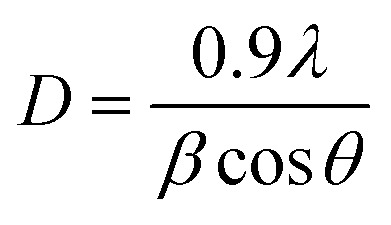


**Fig. 3:**
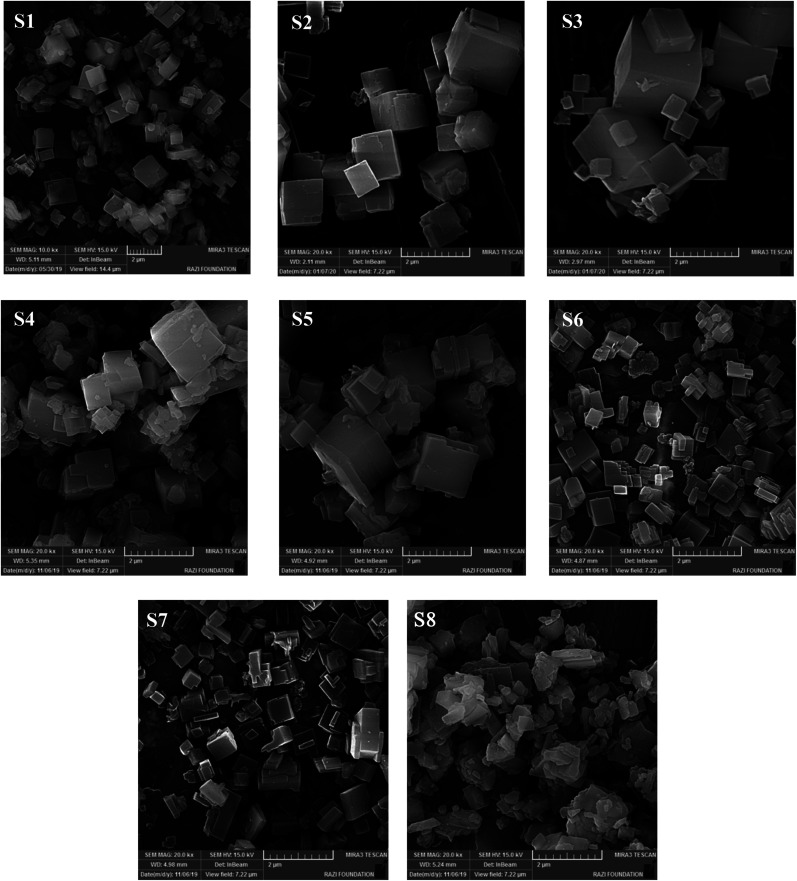
FESEM images of prepared samples.

Fig. 3 illustrates the FESEM images of calcined samples. The S1 sample, which is synthesized without the addition of any seed, has cubic-like morphology and covers a wide range of particle sizes from 0.4–2.1 μm with an average particle size of about 1.1 μm. Regarding the particle sizes in Table 2, the seeding induced catalysts present smaller average particle sizes (of about 0.4–0.85 μm) compared to S1. Indeed, the seeds prepare adequate surface area for the nucleation stage that could control the crystal sizes.^29^ Initial particles can also be generated by dissolved seed and considerably reduce the crystal sizes owing to Ostwald ripening mechanism.^20^

**Table 2:** Results of EDS and SEM of catalysts

Sample no.	The molar composition of the product^a^	The molar composition of the product^b^	Si incorporation (mole)^a^	Average particle size (μm)
S1	Al_0.510_P_0.406_Si_0.114_O_2_	—	0.85	1.1
S2	Al_0.473_P_0.411_Si_0.130_O_2_	Al_0.576_P_0.339_Si_0.085_O_2_	0.98	0.70
S3	Al_0.468_P_0.387_Si_0.164_O_2_	Al_0.573_P_0.345_Si_0.082_O_2_	1.23	0.78
S4	Al_0.481_P_0.400_Si_0.164_O_2_	Al_0.586_P_0.336_Si_0.078_O_2_	1.04	0.79
S5	Al_0.532_P_0.377_Si_0.128_O_2_	—	0.95	0.84
S6	Al_0.505_P_0.408_Si_0.110_O_2_	Al_0.583_P_0.343_Si_0.074_O_2_	0.82	0.41
S7	Al_0.492_P_0.390_Si_0.141_O_2_	Al_0.594_P_0.347_Si_0.060_O_2_	1.06	0.43
S8	Al_0.470_P_0.390_Si_0.159_O_2_	Al_0.583_P_0.337_Si_0.080_O_2_	1.20	—

a Measured based on EDS analysis.

b Measured based on ICP analysis.

Increasing the template concentration indicates that more uniform morphology with slightly smaller average particle sizes can be observed in Fig. 3. As mentioned earlier, the dissolution of seed as well as the higher structure-directing agent cause the generation of more nucleation sites and accelerates supersaturation conditions, which resulted in the generation of smaller particles.^30^ S6, which is prepared with the highest seed synthesis time, has the smallest particles and the most uniform particle sizes distribution compared to S4 and S5. It seems that S2, S3, and S6 have relatively more uniform and smoother external surface in FESEM images, which could be related to their suitable crystallinity, that confirm the XRD results. Among three types of seeds, dried form leads to the creation of smaller particles. Based on the XRD analysis, ALPO_4_-18 phase presents as an impurity in S8, which prevents the evolution of SAPO-34 crystals and/or interferes with the growth of SAPO-34 crystals and resulted in the formation of irregularly shaped particles. Some slice-like ALPO_4_-18 particles or CHA-AEI intergrowths without specific shape are observed in the micrograph of S8 along with SAPO-34 cubic particles. The appearance of disordered morphology also could be related to the presence of amorphous materials that were detected in the XRD pattern of S8.

The energy-dispersive X-ray spectroscopy, which is generally coupled with FESEM, is a technique for determining the elemental composition in semi-quantitative and spatially forms. It should be noted that this analysis is not representative of the bulk composition of the samples. The information of surface chemical compositions is presented in Table 2. According to the reported data, all components in the precursor have existed in the EDS results, which shows that a layer of SAPO-34 is probably formed on the surface of samples.^31^ The Si incorporation greater than 1 could be corresponded to the incorporated intergradient and/or the leftover intergradient on the external surface of particles as an amorphous phase 9,.^32^ Referring to FESEM and XRD, no amorphous phase was detected, except for S8, which revealed that the used elements were incorporated on the external surface of S1 to S7. The Si incorporation that was determined as the ratio of Si/(Si + Al + P)_solid_ to Si/(Si + Al + P)_gel_ is relatively high for S8 that can be related to the remaining amorphous silica on the external surface area.^9^ It is noteworthy to mention that the presence of amorphous material in S8 was discovered by XRD and FESEM analyses.

According to the inductively coupled plasma (ICP) analysis, the information of bulk chemical composition for six samples is listed in Table 2. It can be seen that these samples have relatively similar molar chemical compositions. It can be found that as the concentration of templates decreases, the Si content slightly decreases from S2, S3 to S4. It might be due to the role of organic templates to increase the incorporation of Si in the SAPO-34 framework.^33^ It should be noted that the Si content affects the acidity. Accordingly, the density of acid sites is expected to decreases as the template amount decreases. The higher amount of Si in sample S8 among the samples prepared with a lower concentration of template might be ascribed to the remaining amorphous materials on the extra-framework as confirmed by XRD and FESEM analyses.

**Fig. 4:**
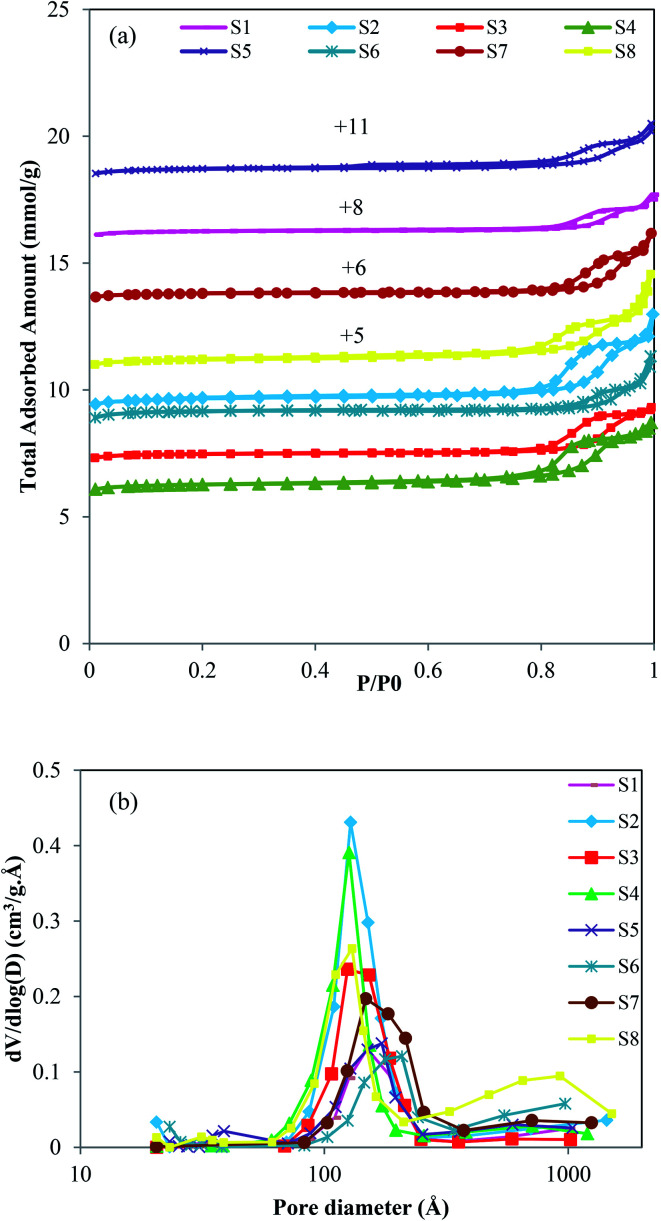
(a) N_2_ adsorption–desorption isotherms and (b) BJH pore size distribution of synthesized samples.

The surface area and pore volumes data of zeolites have been evaluated by N_2_ adsorption/desorption analysis. The obtained data are illustrated in Fig. 4 and are listed in Table 3. It can be seen that all samples have type I and IV isotherms with a hysteresis loop at a relative pressure of about 0.7 < *P*/*P*_0_ < 0.95, informing the existence of mesopores in their structures. As presented in Table 3, the BET (354.13 m^2^ g^−1^) and the external surface (7.96 m^2^ g^−1^) areas of S1 are much less than seeding induced samples that can be due to the higher crystallite and particle sizes of S1. The obtained results indicate that applying seed crystals in the synthesis of samples not only reduces the crystal/particle sizes but also enhances their surface areas and average pore width. As the seed synthesis time decreases from 24 h or 12 h to 6 h, the average pore width increases from 2.1 to 2.5 nm. Furthermore, as the template amount of sample S2 or S3 decreases to S4, the average pore width increases from 2.3 to 2.5 nm. It indicates that the less concentration of template and lower seed synthesis time in sample S4 lead to the formation of pores with the larger sizes. The results show that introducing seed is beneficial for increasing mesopore volume and external surface area. The larger specific surface area of S2 and S6 could be attributed to the combined effects of their small crystallite and particle sizes and their relatively high crystallinity. The smaller surface area and lower micropore volume of S4 and S8 might be due to the existence of SAPO-5 and ALPO-18/SiO_2_ impurities, respectively as well as the defective structure which is confirmed with XRD and FESEM results. The BJH method was carried out in the mesopore section to analyze the mesopore size distribution. As indicated in Fig. 4(b) the pore sizes of all catalysts are centered at around 12–22 nm that confirms the presence of mesopores in their structures. S2 and S4 have narrower size distribution at the diameter of about 12.7 nm, which reveals the higher mesopores created in their structures, as is demonstrated in Table 3. A broad pore size distribution can also be seen for sample S8 in the range of macropores diameter. This result is in good agreement with the highest uptake of S8 at high relative pressure that emphasizes the presence of mesopores or macropores in the structure. This might be due to the inter-crystalline pores created by assembling of the slice-like ALPO-18.^34^

**Table 3:** BET surface analysis of conventional and seeding induced samples

Sample no.	*S*_BET_ a (m^2^ g^−1^)	*S*_micro_ b (m^2^ g^−1^)	*S*_ext_ b (m^2^ g^−1^)	*V*_micro_ c (cm^3^ g^−1^)	*V*_meso_ c (cm^3^ g^−1^)	*d*_p_ d (nm)
S1	354.13	346.16	7.96	0.165	0.011	1.8
S2	718.99	689.05	29.94	0.321	0.098	2.3
S3	555.33	542.18	13.15	0.253	0.061	2.3
S4	466.67	445.07	21.60	0.207	0.083	2.5
S5	573.61	554.93	18.67	0.259	0.047	2.1
S6	682.23	658.93	23.29	0.307	0.048	2.1
S7	580.05	565.66	14.39	0.264	0.065	2.2
S8	461.22	438.50	22.72	0.204	0.077	2.4

a The BET surface area was measured applying the Brunauer–Emmett–Teller (BET) equation.

b The external surface area (*S*_ext_) was evaluated from the *t*-plot method. *S*_micro_ was obtained by subtracting *S*_ext_ from *S*_BET_.

c The micropore volume (*V*_micro_) was calculated using the *t*-plot method, *V*_meso_ = *V*_tot_ − *V*_micro_, in which the *V*_total_ calculated from adsorbed amount at *P*/*P*_0_ = 0.975.

d The average pore width (*d*_p_) was measured based on the BET.

In conclusion, the BET surface area and the micropore volume drop as the template amount decreases. Moreover, these parameters decline as the seed synthesis time increases from 6 h to 24 h. Although there is no significant difference in the BET surface area of samples prepared with the solution and dried seed, the calcined one has a lower surface area.

**Fig. 5:**
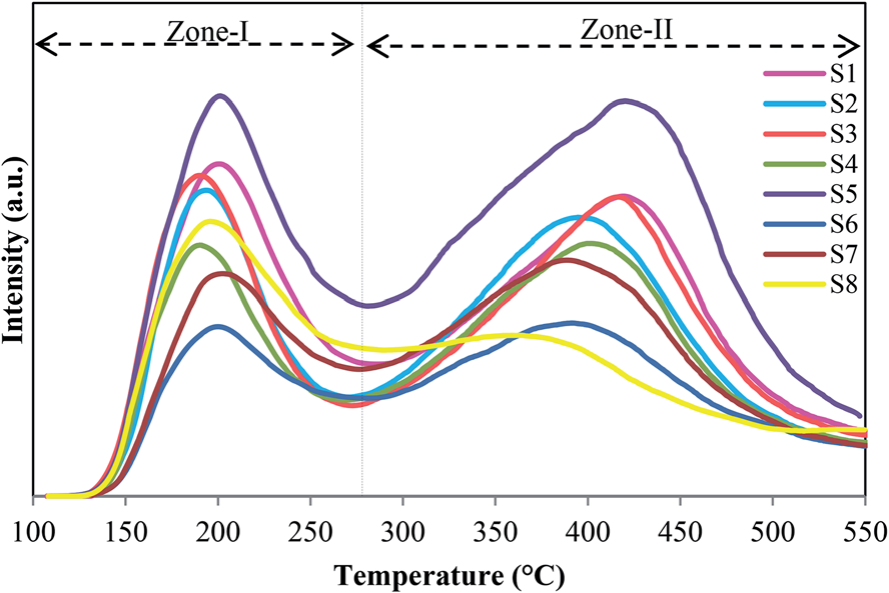
NH_3_-TPD profiles of synthesized samples.

The acidic properties of calcined samples have been characterized by NH_3_-TPD. The desorption curves are drawn in Fig. 5, and the values of weak and strong acid sites with the peak positions are presented in Table 4. As illustrated in Fig. 5, all samples have two distinct peaks centered at around 190–210 °C and 350–430 °C, which are assigned to weak and strong acid sites, respectively. In particular, the former located in Zone-I is related to the desorption of physisorbed ammonia and NH_3_ adsorbed on structural OH group's defects (*e.g.*, P, Si, Al–OH), while the latter located in Zone-II is mainly relevant to the desorption of NH_3_ from strong Brønsted acid sites that are recognized as the main active sites for MTO process.^22,35^ It has been reported that the strong acid sites of SAPOs are generated by incorporation of Si in the ALPO framework by two mechanisms (SM2 and SM3). The Si(4Al) structure is driven from SM2 mechanism, in which one Si is substituted for one P, which leads to the formation of weaker Brønsted acid sites. Si-rich regions of the type Si(0–3Al) within the SAPO-34 structure are generated by SM3, in which a pair of adjacent Al and P are substituted by two Si atoms, resulting in the stronger acid sites.^36^ Regarding the desorption curves of ammonia, S5 has the maximum amount of weak and strong acid sites and the highest strength in the strong acid sites among all catalysts. It might be due to the high amount of Si(0–3Al) structures.^36^ In comparison, sample S8 exhibits the lowest concentration and strength in the strong acid sites. It can be attributed to the presence of ALPO_4_-18 as an impurity, which is electrically neutral and possesses very weak acidity. Furthermore, structural defects, confirmed by XRD and FESEM results, maybe another reason for decreasing the acidity of this sample. Consequently, the concentration of strong acid sites decreases slightly with the reduction of template amount due to the decrease of Si content confirmed by ICP analysis. The low concentration of S4 contaminated with SAPO-5 can be ascribed to the inaccessibility of hydroxyls in the six-ring channels of AFI structure.^37^ It can be seen that samples prepared by dried (S7) and calcined (S8) seeds exhibit lower acidity in comparison with S5. It might result from the existence of ALPO_4_-18 in S7 and S8 as confirmed by the XRD pattern earlier.

**Table 4:** Acidity results determined with NH_3_-TPD

Sample no.	Acid amount (mmol g^−1^)			Max peak temperature	
	Weak	Strong	Total acidity	*T*_P_1__	*T*_P_2__
S1	0.32	0.49	0.81	201	419
S2	0.31	0.55	0.87	193	394
S3	0.34	0.54	0.89	189	416
S4	0.29	0.49	0.78	190	400
S5	0.49	0.88	1.37	201	420
S6	0.34	0.60	0.94	199	390
S7	0.28	0.50	0.78	202	388
S8	0.36	0.38	0.74	195	359

It can be conveyed that seed synthesis conditions (time and type), as well as the template amount, affect the mechanism of Si incorporation in the ALPO_4_ lattice during the preparation of SAPO-34, which leads to catalysts with different acidity.

## 6. Catalytic performance in the MTO reaction

**Fig. 6:**
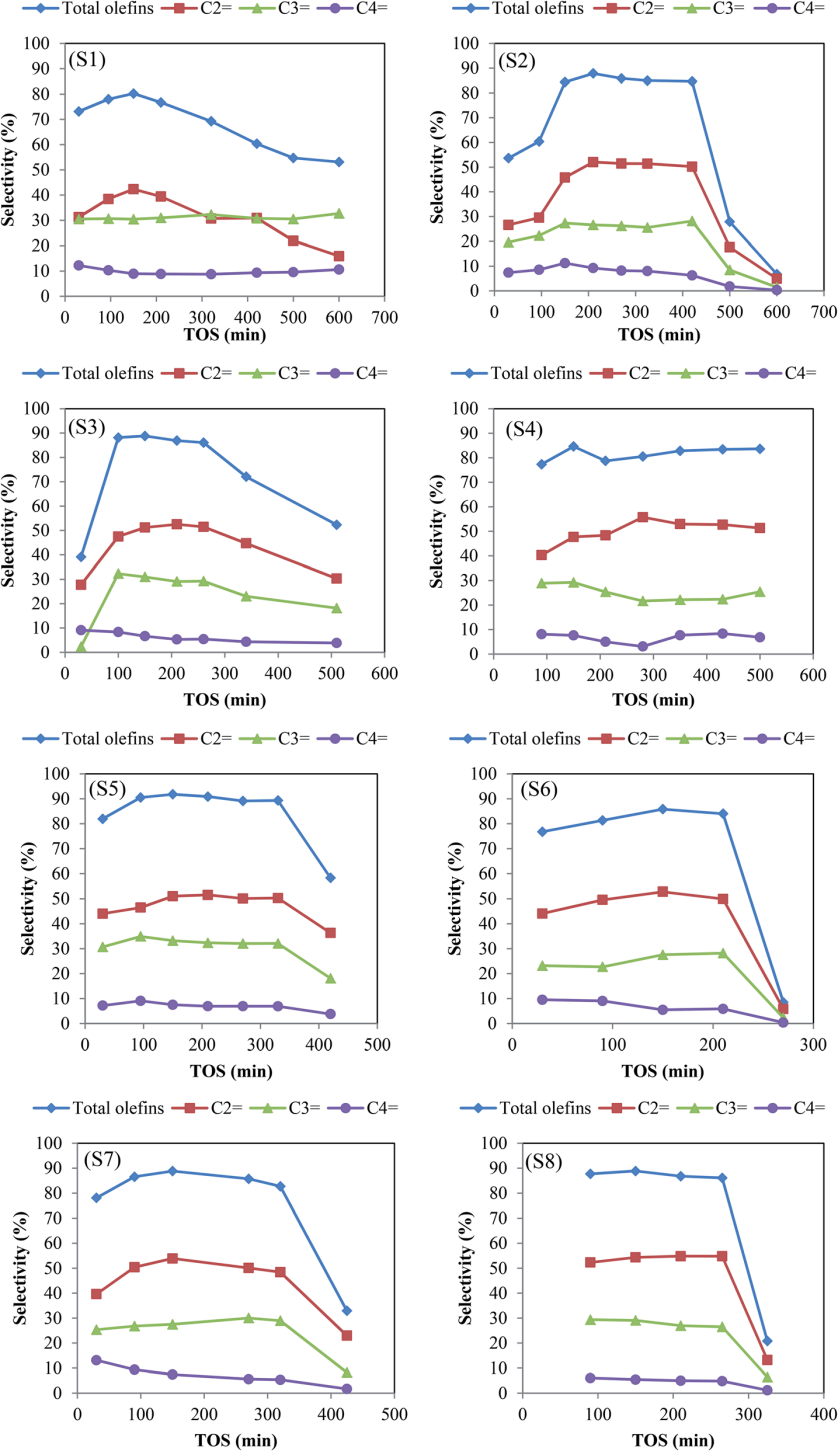
The selectivity of olefins over prepared samples. Experimental conditions: *T* = 425 °C, WHSV of 2 h^−1^, catalyst weight = 2 g.

The catalytic performance of the samples was evaluated in the methanol to olefin process under severe feed conditions (72 wt% methanol in water) and at 425 °C; to achieve the most realistic results. For all SAPO-34 samples, the methanol conversion of above 98% was achieved in the process. The product distribution of the samples with time on stream (TOS) is illustrated in Fig. 6. The seed-assisted samples had higher total light olefins selectivity compared to the conventional catalyst, especially for the S5 sample, in which template consumption decreased by 40% in comparison with S1. More light olefins selectivity of seeding induced samples results from particle and crystallite size reduction and the increase of BET and external surface areas as well as the enhancement of mesopore volume. As the particle sizes and crystallite sizes decrease, the diffusion path lengths and the coke formation reduce. Subsequently, the light olefins can more easily be removed from the catalyst pore structure before being converted to aromatics and paraffins by hydrogen transfer reaction and cyclization.^22^ In addition, the existence of intra-crystalline mesopores leads to a shorter diffusion path length, better accessibility to the acidic sites, and easier diffusion of reactants and products *via* mesopores. It can be clearly seen that the amount of templates, the type of seeds, and the seed synthesis time alter the olefins selectivity and catalytic lifetime.

The S4, S5, S6 samples that are prepared with different seed synthesis times showed the maximum olefins selectivity of 84.6%, 91.8%, and 85.8%, respectively. The higher olefins selectivity of S5 not only related to its more appropriate acidity but also can be attributed to its small crystallite size, which is confirmed by the XRD pattern. The lifetime of these samples decreases as follows: S4 (>500 min) > S5 (330 min) > S6 (210 min). It can be seen that as the seed synthesis time increases the catalyst lifetime decreases. S4 exhibited the highest activity during reaction time. Despite the presence of SAPO-5 impurity, the total olefins selectivity of above 80% was retained for more than 500 min, which is at least triple higher than that of conventional one. This result can be attributed to mesopores created in its structure as well as appropriate acidity (density and strength) that is followed by the TPD and BET results.

Although small crystals are desirable for samples because of large external surfaces and short diffusion paths, very small crystals can decline the catalytic performance. The large external surface area of the extremely small particles limits the inner surface areas and reduces the number of available acid sites in the micropores that act as active sites.^38^ Accordingly, the rapid deactivation of S6 might be related to its small particles with a higher external surface area observed in FESEM and BET analyses. It is noteworthy to mention that using industrial feed condition (72% methanol in water) resulted in the earlier catalyst deactivation as well as lower olefins production.

Two reasons have been reported for the deactivation of SAPO-34 catalysts. First, the deactivation occurs when the bulky hydrocarbons are trapped in the small cages of catalysts and form coke species. Second, the deactivation related to the gradual loss of active sites with MTO reaction progress.^39^ In other words, as the reaction proceeds, the deposition of coke species on the acidic sites not only led to the loss of activity of acidic sites but also restricts the access of reactant and product molecules to the other active sites, which resulted in the reduction of main products selectivity at high TOS. Hence, the superior performance of S5 in the MTO reaction can be justified by its high amount of active sites in accordance with TPD analysis. It should be noted that with increasing acidity in the appropriate range, the selectivity of light olefins increases.^40^ Compared to the conventional sample, the total olefins selectivity of S5 (91.8%) has been improved by about 14.43%.

The S2, S3 and S4 samples that are prepared with different amounts of template demonstrated maximum olefins selectivity of 87.84%, 88.80% and 84.61%, respectively. The order of these samples lifetime decreases as follows: S4 (>500 min) > S2 (420 min) > S3 (260 min). These results could be elucidated by BET and external surface areas as well as the concentration and the strength of strong acid sites. The lower stability of S3 might be attributed to its higher strong acid strength and less external surface area.

Study the type of seeds indicated that the solution and dried seed are more effective on catalytic performance. Although S7 and S8 had the same maximum olefins selectivity, the activity of S8 (265 min) dropped faster than S7 (320 min). It is possible that an amount of template exists in the solution and dried seed improves the CHA phase purity and the total specific surface area of S5 and S7, which results in higher catalytic performance. In addition, the higher appropriate acidity of these two samples is another reason for their better MTO performance.

The details of product distribution plus catalysts lifetime are given in Table 5. The maximum selectivity of ethylene is higher than propylene for all samples. It could be resulted from the more reactivity of propylene and butene compared to ethylene, which causes the oligomerization of them to bigger molecules that are confined in SAPO-34 cages and consequently are cracked to ethylene at the reaction temperature of 425 °C (>400 °C). In other words, it is accepted that light olefins are generated *via* hydrocarbon pool mechanism (HCP), in which the aromatic-based species are trapped in the zeolite cages and act as active intermediates.^41^ C^=^_2_ and C^=^_3_ are the first products that can undergo secondary reactions and generate the bulky hydrocarbons that eventually cracked to ethylene.^9^

**Table 5:** Lifetime and product selectivity of samples in the MTO reaction (WHSV = 2 h^−1^, *T* = 425 °C, %wt MeOH : H_2_O = 72 : 28)^a^^,^^b^

Sample		Lifetime (min)	Selectivity (%)								
			CH_4_	C^=^_2_	C^=^_3_	C^=^_4_	Total olefins	C_2_–C_4_	DME	C5+	CO + CO_2_
S1	*t* = 150*	150	3.22	41.48	29.80	8.92	80.21	4.52	0	9.46	2.11
	*t* = 325		6.05	30.01	29.89	9.34	69.25	3.76	0.20	16.45	4.12
	*t* = 500		9.51	14.41	29.68	10.59	54.70	4.75	0	22.79	7.60
S2	*t* = 210*	420	3.58	52.02	26.59	9.23	87.84	2.53	0	5.40	0.09
	*t* = 420		4.26	50.18	28.17	6.28	84.65	1.33	2.08	6.05	0.19
	*t* = 510		6.27	17.62	8.44	1.78	27.85	0.52	61.24	2.03	0.72
S3	*t* = 150*	260	2.86	51.18	30.97	6.64	88.80	3.63	1.36	3.15	0.11
	*t* = 260		3.18	51.44	29.24	5.40	86.09	2.30	5.91	2.02	0.13
	*t* = 510		2.54	30.30	18.15	3.82	52.29	0.84	42.86	0.83	0.13
S4	*t* = 150*	500	3.89	47.71	29.25	7.64	84.61	5.58	0	3.38	0.86
	*t* = 280		4.86	55.74	21.67	3.11	80.53	8.06	0	5.24	0.91
	*t* = 500		5.32	51.32	25.44	6.86	83.63	7.64	1.71	1.14	0
S5	*t* = 150*	330	2.38	51.03	33.22	7.52	91.80	1.32	0	4.02	0.26
	*t* = 330		3.53	50.30	32.09	6.91	89.31	1.09	0	4.17	0.44
	*t* = 420		18.16	36.33	18.12	3.81	58.27	4.05	0	9.80	9.60
S6	*t* = 150*	210	7.95	52.81	27.56	5.47	85.85	1.58	0	2.59	1.38
	*t* = 270		9.06	5.83	2.29	0.40	8.52	1.30	77.53	0.76	2.79
S7	*t* = 150*	320	3.28	53.91	27.48	7.45	88.85	0.88	0	6.13	0.36
	*t* = 320		5.68	48.43	28.95	5.35	82.73	0.70	0	9.17	0.98
	*t* = 425		15.36	22.98	8.24	1.71	32.93	1.33	0	40.04	8.46
S8	*t* = 150*	265	6.88	54.35	29.05	5.43	88.85	1.38	0	1.84	0.26
	*t* = 265		8.16	54.79	26.47	4.82	86.09	0.97	1.59	2.30	0.23
	*t* = 325		6.68	13.22	6.40	1.17	20.80	0.55	70.62	0.50	0.75

a Catalyst lifetime is defined as the reaction duration before the olefins selectivity drop down.

b The selectivity of products at different times. The time of the highest selectivity is marked with a star.

Among seeding induced samples, S6 shows the shortest lifetime of 210 min, and the maximum selectivity of 85.85% for total light olefins. The selectivity dropped sharply to 8.52% at a reaction time of 270 min. Although the catalyst activity of S1 began to decline after 150 min, its selectivity decreased slightly and reached 53.11% after 600 min, which could be due to higher crystallinity and the lower concentration of strong acid sites that might lessen the coke deposition.

Table 6 represents the comparison of our catalytic performance in the MTO process with those SAPO-34 catalysts synthesized *via* a seed-assisted method by other researchers. It can be seen that the performance of our synthesized samples, with a significant reduction of template concentration, was better than the performance of catalysts from similar studies. It is important to note that our superior results were obtained not only with a notable decrease of template content but also the reactor tests were performed under the industrial feed composition.

**Table 6:** Catalytic performance results of SAPO-34 samples synthesized by seed-assisted method

Reference	Template (based on Al_2_O_3_, mol)	Lifetime (min)	Olefins selectivity	Operating conditions	Lifetime definition
Gao *et al.*^42^	TEA = 1.8 & TEABr = 1.5	242	85.2% (C_2_–C_3_)	*T* = 450 °C	Conv > 99%
				WHSV = 4.16 h^−1^	
Chen *et al.*^21^	MOR = 4	166	84.3% (C_2_–C_3_)	*T* = 420 °C	Conv = 100%
				WHSV = 4 h^−1^	
Sun *et al.*^22^	MOR = 4	264	83.6% (C_2_–C_3_)	*T* = 425 °C	Conv > 99%
				WHSV = 2 h^−1^	
Eslami *et al.*^29^	TEAOH = 2	—	15% (C_2_–C_3_)	*T* = 400 °C	—
				GHSV = 4200 h^−1^	
Sun *et al.*^34^	TEAOH = 0.94 & TEA = 1.56	396	83% (C_2_–C_3_)	*T* = 425 °C	Conv > 99%
				WHSV = 2 h^−1^	
Lyu *et al.*^24^	TEAOH = 1	330	83.6% (C_2_–C_4_)	*T* = 400 °C	Conv > 95%
				WHSV = 0.5 h^−1^	
Lu *et al.*^23^	MOR = 1	140	—	*T* = 400 °C	Conv > 99%
				WHSV = 3 h^−1^	
Present research	TEAOH = 0.6 & MOR = 0.6	330, > 500	91.8% (C_2_–C_4_), 84.6% (C_2_–C_4_)	*T* = 425 °C	Conv > 98%
				WHSV = 2 h^−1^	
				MeOH : H_2_O = 72 wt%	

In general, SAPO-34 with less acidic concentration and strength shows slower catalyst deactivation and superior MTO catalytic performance. Nevertheless, samples with the least acidic sites necessarily do not possess the longest lifetime. The accessibility to acidic sites might be slightly hindered by coke deposition, leading to quicker deactivation. Accordingly, to attain optimal MTO efficiency for the SAPO-34 zeolite, it is reasonable to adjust both mesoporosity and microporosity, and regulate the acidity, accurately. As a result, sample S5 showed better catalytic performance than other samples, especially in terms of olefins selectivity, due to its higher suitable acidity, small crystallite size, large surface areas, and appropriate particle size.

## 7. Conclusions

In summary, we investigated the effect of seed synthesis time and three types of seeds (solution, dried, and calcined) on the physicochemical properties and the catalytic performance of SAPO-34 zeolites in the MTO reaction. Pure SAPO-34 was obtained with consuming 40% less template by applying 12 h solution seed. The resultant sample had a longer lifetime (330 min) and higher total olefins selectivity (91.79%) compared to the conventional sample, which shows the improvement of 14.43% in overall catalysts performance. The seeding induced catalysts exhibited smaller crystallite/particle sizes and higher BET and external surface areas as well as more mesopore volume compared to the unseeded catalyst, resulted in the higher catalyst performance. It was indicated that the seed synthesis conditions affect the acidity of zeolites and must be controlled to obtain optimal catalytic performance. Excellent catalytic performance of samples accompanied by the notable reducing template consumption provide a promising future for a cost-effective and more eco-friendly green route of synthesizing zeolites *via* a seeding method.

## Conflicts of interest

The authors declare that they have no conflict of interest.

## Supplementary Material
